# Electroacupuncture for the Prevention of Postoperative Cognitive Dysfunction Among Older Adults Undergoing Hip and Knee Arthroplasty: A Systematic Review and Meta-Analysis of Randomized Controlled Trials

**DOI:** 10.3389/fmed.2021.778474

**Published:** 2022-01-04

**Authors:** Liang Ou, Zhen Shen, Tiantian Zhang, Zehua Chen, Lin Zhang, Daoqing Xu, Dezhong Kong, Qi Qi, Yanchang Huang, Weichen Huang, Yingfu Meng

**Affiliations:** ^1^Department of Orthopedics, The Second Affiliated Hospital of Guizhou University of Chinese Medicine, Guiyang, China; ^2^Department of Orthopedics, Kunming Municipal Hospital of Traditional Chinese Medicine, The Third Affiliated Hospital of Yunnan University of Chinese Medicine, Kunming, China; ^3^The Graduate School, Hunan University of Chinese Medicine, Changsha, China; ^4^The Fifth Clinical Medical College of Guangzhou University of Chinese Medicine, Guangzhou, China

**Keywords:** electroacupuncture, postoperative cognitive dysfunction, arthroplasty, prevention, elderly, meta-analysis, systematic review

## Abstract

**Background:** Postoperative cognitive dysfunction (POCD) is a common surgical complication in elderly patients undergoing hip and knee replacement. Electroacupuncture (EA) may have a protective effect on postoperative cognitive function, but relevant evidence remains uncertain.

**Objective:** To systematically evaluate the evidence of EA for the prevention of POCD after total joint arthroplasty.

**Methods:** PubMed, Embase, Cochrane Central Register of Controlled Trials (CENTRAL), China National Knowledge Infrastructure (CNKI), Wanfang Data, VIP, and Chinese Biomedical Literature Database (CBM) databases were searched until May 1, 2021. Randomized controlled trials (RCTs) in which patients undergoing hip and knee replacement pretreated with EA for preventing POCD were included. The risk of bias was assessed by the Cochrane Collaboration tool. Meta-analysis was performed using Review Manager version 5.4.

**Results:** A total of 11 RCTs with 949 patients were identified. Meta-analysis showed that compared with controls, EA pretreatment significantly reduced the incidence of POCD at 1, 3, and 7 days and 3 and 6 months after the operation. EA was also superior in improving the Mini-Mental State Examination (MMSE) scores on the third postoperative day, but not on the first postoperative day. Neuron-specific enolase (NSE) and interleukin-1β (IL-1β) in the EA group were significantly lower than that in the control group. There was no difference in S100β between the EA group and the control group. Compared to the control group, tumor necrosis factor-α (TNF-α) levels were not significantly lower in the EA group at postoperative hour 0, while significantly decreased at postoperative hours 24 and 48.

**Conclusion:** Our results suggest that EA pretreatment is an effective adjunctive therapy for reducing the incidence of POCD for patients receiving total joint replacement surgery. Its effect was embodied in improving the MMSE scores and NSE, IL-1β, and TNF-α levels, whereas it had no significant effect on S100β levels. Meanwhile, the benefits of EA for improving POCD need further strengthening and support from more large-scale, high-quality, and good-homogeneity RCTs.

**Systematic Review Registration:** https://osf.io/xb3e8.

## Introduction

Postoperative cognitive dysfunction (POCD) is a common complication in elderly surgical patients, which seriously threatens early rehabilitation after surgery of patients and their long-term quality of life (1, 2). With the deepening of the aging of society, the number of older adults who are undergoing anesthesia and surgery is increasing, which will attribute to a high frequency of POCD. Meanwhile, additional diseases and poor basal functional status of elderly patients lead to an increased incidence of POCD (3). As previously reported, the prevalence of POCD ranged from 25 to 40% among elderly patients (4). POCD is a neurological dysfunction, characterized by the continuous decline of cognitive performance after surgical anesthesia, including disturbances in consciousness, orientation, thinking, memory, and executive function. Furthermore, studies have found that POCD could potentially result in an increased risk of some neurodegenerative diseases, including Alzheimer's disease and Parkinson's disease (5–7). However, POCD remains difficult to treat effectively, due to the pathophysiology and risk factors that are incompletely understood (8).

Cognitive dysfunction following hip and knee joint replacement surgery has received a great deal of attention during the last few decades (9). Once a POCD occurs, it means that postoperative rehabilitation will be delayed and hospital stay will be prolonged for them. In more serious cases, POCD would directly or indirectly lead to surgery failure. Apparently, these consequences contradict the concept of enhanced recovery after surgery that aims to have the most favorable treatment course for the patient after surgery (10). Taking into consideration that no effective treatment for POCD has been currently developed, we should emphasize the importance of prevention strategies. In other words, elderly patients undergoing hip and knee joint replacement surgery could be treated with preoperative intervention to decrease the risk of POCD.

Electroacupuncture (EA) is an improvement method combining acupuncture and electric stimulation, which uses an electrical device connected to a needle to send electrical currents to the acupoint and, thereby, enhance the stimulation. When the acupuncture point has a sense of qi, the therapist selects the wave type, slowly adjusts to the required output current, and performs electrical stimulation generally for 5–20 min. Numerous studies have demonstrated the cognitive improvement effects of EA stimulation over the past several years (11, 12). EA applied before surgery can effectively reduce the dose of narcotic drugs, improve stress response, reduce the occurrence of postsurgical complications, and promote functional rehabilitation in patients undergoing surgery (13, 14). Thus, EA has been proposed to be used for the treatment of various kinds of neurological disorders, including POCD. Recently, there are a growing number of studies focusing on preventing and treating POCD with EA, especially for patients receiving joint replacement surgery. However, to the best of our knowledge, no meta-analysis has as yet evaluated the effects of EA for the prevention of POCD among older adults undergoing hip and knee arthroplasty. Consequently, this meta-analysis of randomized controlled trials (RCTs) was performed to summarize the available evidence to investigate the preventive therapeutic efficacy of EA on POCD for them.

## Methods

Ethical approval was not provided in this study because all the analyses were based on previously published studies. This study was registered in the Open Science Framework (https://osf.io/XB3E8) and performed in accordance with the Preferred Reporting Items for Systematic Reviews and Meta-Analyses (PRISMA) guideline (15).

### Research Strategy

Potentially relevant studies were searched in PubMed, Embase, Cochrane Central Register of Controlled Trials (CENTRAL), China National Knowledge Infrastructure (CNKI), Wanfang Data, Chinese Scientific Journal Data (VIP), and Chinese Biomedical Literature Database (CBM), up to May 1, 2021. Search terms, such as “electroacupuncture,” “arthroplasty,” “joint replacement,” “total knee arthroplasty (TKA),” “total hip arthroplasty (THA),” “postoperative cognitive dysfunction,” “POCD,” and “randomized controlled trial”, were used to retrieve. Detailed search strategies are given in Supplementary Figure 1. Meanwhile, we added a manual search for references of included studies.

### Eligibility Criteria

Randomized controlled trials related to using EA for the prevention of POCD after hip and knee replacement in elderly patients were included. The included studies should meet all of the following criteria: (1) Patients: patients (>60 years old) undergoing primary THA and TKA and without cognitive dysfunction before intervention; (2) Intervention: EA; (3) Comparators: EA vs. blank control, EA vs. sham EA or placebo; (4) Outcomes: the incidence of POCD as the main evaluation index to be reported and secondary outcomes such as the Mini-Mental State Examination (MMSE) scores, neuron-specific enolase (NSE), S100β, interleukin-1β (IL-1β), and tumor necrosis factor-α (TNF-α) to be reported selectively; and (5) Study design: a clinical randomized controlled study published in Chinese or English. Studies would be excluded if met any of the following criteria: observational studies, protocols, case reports, animal experimental studies, reviews, duplicated publications, and full-text unavailable articles.

### Data Extraction

First, two reviewers (LO and TZ) independently went through the standard literature screening process: removing duplicate studies, eliminating obviously irrelevant studies by reading the titles and abstracts, and including eligibility studies by reading the full text. Second, we used a standardized form to independently extract the main information from included articles including primary author, publication year, age and gender of patients, sample size, type of anesthesia, type of operation, intervention type, intervention parameters, and outcomes. Finally, disagreement in the above process, if any, would be resolved through discussion or consultation with the third reviewer (ZS).

### Quality Assessment

Adhering to the standards advised by the Cochrane Collaboration risk of bias table (16), two investigators (LO and TZ) assessed the methodological quality of all the included pieces of literature independently. Any inconsistency was resolved by discussion with the third author (ZS). The risk of bias for each study was evaluated from the following seven aspects: random sequence generation, allocation concealment, blinding of participants and personnel, blinding of outcome assessment, the integrity of results data, selective reporting of results, and other biases. The assessment result of each item is classified into three categories: high, unclear, and low risk.

### Statistical Analysis

The Review Manager version 5.4 software (2020) (Cochrane, UK) provided by the Cochrane Collaboration was used to conduct all the meta-analyses of the observation outcomes in the included studies, and the results were illustrated by the forest map intuitively. In this study, continuous variables were pooled by mean difference (MD) and dichotomous variables were pooled by odds ratio (OR). All the pooled effects were expressed with 95% CI. Heterogeneity was tested by Cochran's Q test and *I*^2^ statistic. An *I*^2^ statistic >50% represented high heterogeneity. Subsequently, subgroup analysis or sensitivity analysis would be applied to investigate the sources of heterogeneity, when there was substantially heterogeneous. The random-effects or fixed-effects models were selected depending on the heterogeneity. Publication bias was estimated by Begg's test and Egger's test, which are conducted by using the Stata 14 (2015) (StataCorp LP, College Station, TX, USA). Value of *p* < 0.05 was considered to be statistically significant.

## Results

### Study Identification and Selection

**Figure 1:**
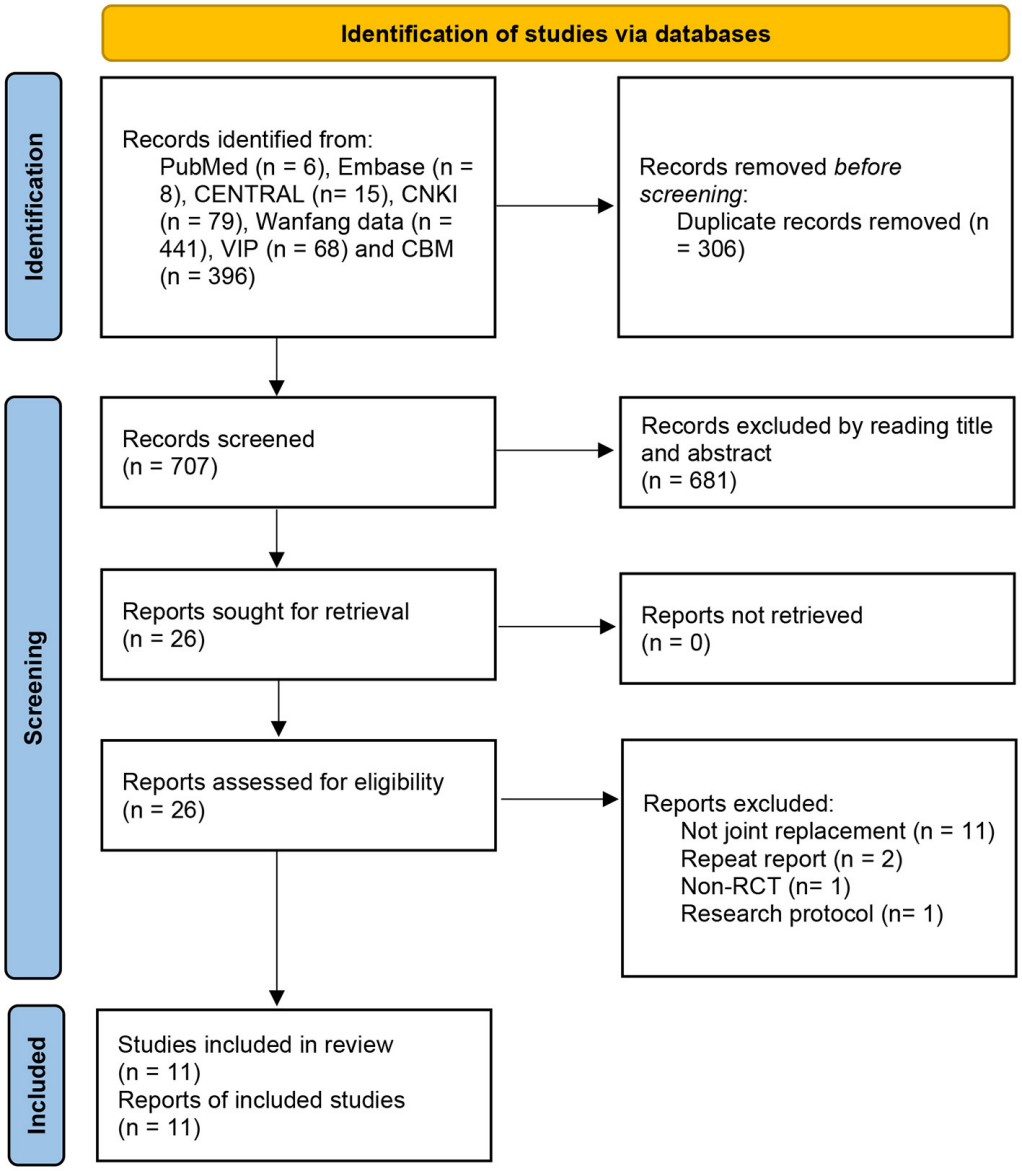
Flow diagram of literature search.

A total of 1,013 related clinical trials were identified by the search strategy and imported into EndNote X8 to remove duplicate literature. After the removal of 306 repeated studies and excluding 684 studies through the title and abstract screening, leaving 26 full-text articles were reviewed and 15 of them were excluded. A total of 11 trials (17–27) met inclusion criteria and were selected for the analysis. The detailed selection flowchart is shown in Figure 1.

### Characteristics of Included Trials

All the included trials were conducted in China, and 949 patients who were over 60 years old were enrolled. Of the included 11 clinical trials, 9 studies (17–21, 23, 25–27) were given the method of general anesthesia and 2 trials (22, 24) were carried out under spinal-epidural anesthesia. In terms of operation type, THA and TKA were with 7 (17, 19–24) and 2 trials (18, 26), respectively. In addition, two trials (25, 27) combined the THA and TKA together for study. The vast majority of trials (*n* = 10) compared EA with blank and only one study (18) compared EA with sham EA. The stimulus frequency and duration of EA treatment differed among studies. The most commonly used acupoints were “Baihui” (DU20) and “Shenting” (DU24). Study characteristics are shown specifically in Table 1.

**Table 1:** Characteristics of the included randomized clinical trials.

**References**	**Age (years)**		**Gender (male/** **female)**		**Sample** **size**		**Operation type**	**Type of anesthesia/anesthesia drugs**	**Intervention**			**Outcomes (time points for evaluation)**
	**EG**	**CG**	**EG**	**CG**	**EG**	**CG**			**EG**	**CG**	**Acupoints/stimulation parameter/time**	
Li et al. (22)	77.2 ± 3.0	76.8 ± 2.7	16/26	14/28	42	42	THA	Spinal-epidural anesthesia/bupivacaine, lidocaine, midazolam	EA	Blank	MS1, MS5/continuous wave, 200 HZ/after anesthesia, through completion of surgery	Incidence of POCD (on 3 d, 7 d, 3 m), NSE (on 0 h, 24 h, 48 h), S-100 β (on 0 h, 24 h, 48 h), AE
Wang et al. (24)	>70	>70	NR	NR	40	40	THA	Spinal-epidural anesthesia/bupivacaine, lidocaine, midazolam	EA	Blank	MS1, MS5, MS7, MS10/continuous wave, 200 HZ/after anesthesia, through completion of surgery	Incidence of POCD (on 3 d, 7 d, 3 m), AE
Zhao et al. (18)	65.2 ± 4.0	66.7 ± 3.8	12/18	14/16	30	30	TKA	General anesthesia/fentanyl, midazolam, propofol, cisatracurium besilate, remifentanil	EA	Sham EA	EX-HN1, DU24, DU20, GB13, LI4, LR3/dilatational wave, 2/100 Hz, 3 mA/30 min, once daily, and 5 days consecutively prior to the surgery	Incidence of POCD (on 1 d, 3 d), MMSE (on 24 h, 72 h), S-100 β (on 24 h, 72 h), IL-1 β (on 24 h, 72 h), TNF-α (on 24 h, 72 h), AE
Jiang et al. (25)	70.2 ± 4.5	71.2 ± 4.8	21/22	20/25	43	45	THA/TKA	General anesthesia/midazolam, propofol, rocuronium bromide, sufentanil, remifentanil	EA	Blank	DU20, DUI4/2-15 Hz/30 min, once daily, and 5 days consecutively prior to the surgery	Incidence of POCD (on 1 d, 7 d)
Liu et al. (19)	65 ± 8	66 ± 5	25/35	26/34	60	60	THA	General anesthesia/fentanyl, midazolam, propofol, cisatracurium besilate	EA	Blank	LI4, LR3/dilatational wave, 2/10 Hz/30 min, once daily, 3 days prior to the surgery and 3 days after surgery, and 30 min before anesthesia, through completion of surgery	Incidence of POCD (on 3 d), MMSE (on 72 h), IL-1 β (on 0 h, 24 h), TNF-α (on oh, 24 h)
Liu et al. (20)	66 ± 7	67 ± 6	16/24	17/23	40	40	THA	General anesthesia/fentanyl, midazolam, propofol, cisatracurium besilate, remifentanil	EA	Blank	LI4, LR3/dilatational wave, 2/10 Hz/30 min, once daily, 3 days prior to the surgery and 3 days after surgery, and 30 min before anesthesia, through completion of surgery	Incidence of POCD (on 3 d), MMSE (on 72 h), NSE (on 0 h, 24 h, 48 h), S-100 β (on 0 h, 24 h, 48 h, 72 h), IL-1 β (on 0 h, 24 h, 72 h), TNF-α (on 0 h, 24 h, 72 h)
Xie et al. (23)	71.9 ± 7.2	70.8 ± 7.7	42/18	37/23	60	60	THA	General anesthesia/midazolam, propofol, fentanyl, cisatracurium besilate, sevoflurane, remifentanil	EA	Blank	GB13, DU24/continuous wave/30 min before completion of surgery, and 30 min, once daily, 2 days after surgery	Incidence of POCD (on 1 d, 3 d, 7 d)
Xu et al. (17)	77.0 ± 6.3	78.4 ± 5.5	32/18	29/21	50	50	THA	General anesthesia/midazolam, remifentanil, propofol, rocuronium bromide, sufentanil	EA	Blank	LI4, LR3/dilatational wave, 2/10 Hz/30 min before anesthesia, through completion of surgery	Incidence of POCD (on 1 d, 3 d, 7 d), MMSE (on 24 h, 72 h)
Tao et al. (21)	72.5 ± 6.7	73.3 ± 5.90	15/15	14/16	30	30	THA	General anesthesia/midazolam, sufentanil, cisatracurium besilate, propofol, sevoflurane	EA	Blank	DU20, PC6, DU24/dilatational wave, 2–15 Hz/after anesthesia, through completion of surgery	Incidence of POCD (on 1d, 7d)
Zhang et al. (27)	74.2 ± 5.9	75.0 ± 6.5	19/22	20/27	41	47	THA/TKA	General anesthesia/midazolam, sufentanil, vecuronium, propofol, isoflurane	EA	Blank	DU20, DU24/dilatational wave, 2–15 Hz/30 min before anesthesia, through completion of surgery	Incidence of POCD (on 1 d, 7 d), S-100 β (on 0 h, 24 h), AE
Zhang et al. (26)	67.5 ± 4.7	70.1 ± 5.3	15/19	20/25	34	35	TKA	General anesthesia/sufentanil, cisatracurium besilate, propofol, isoflurane	EA	Blank	DU20, DU24/dilatational wave, 2–15 Hz/30 min after anesthesia	Incidence of POCD (on 1 d, 7 d), S-100 β (on 0 h, 24 h), IL-1 β (on 0 h, 24 h), TNF-α (on 0 h, 24 h)

*EG, electroacupuncture group; CG, control group; THA, total hip arthroplasty; TKA, total knee arthroplasty; POCD, postoperative cognitive dysfunction; AE, adverse event; NR, No record*.

### Risk of Bias

**Figure 2:**
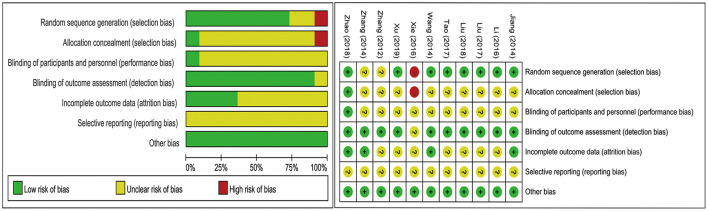
Risk of bias graph.

A total of seven trials (17, 19–22, 24, 25) were randomized in accordance with the random number table and one study (18) used a computer-generated random list. Two trials (26, 27) mentioned random without any method and the remaining one article (23) selected an incorrect randomization with the order of operation time. The allocation concealment processes used in studies were unclear, except that one study (18) used an opaque envelope to seal random numbers. Blind methods for participants were detailed reported in one study (18), which used sham EA as a control. Detection bias performed well in most of the included studies and the dropout rate was reported in four articles (18, 24–26). Because of the insufficient information provided, selective reporting could not be judged in all the studies. Other biases were assessed to be of low risk in all the included trials. An overview of the risk of bias assessment of the included studies is shown in Figure 2.

### Meta-Analysis

#### Incidence of POCD

**Figure 3:**
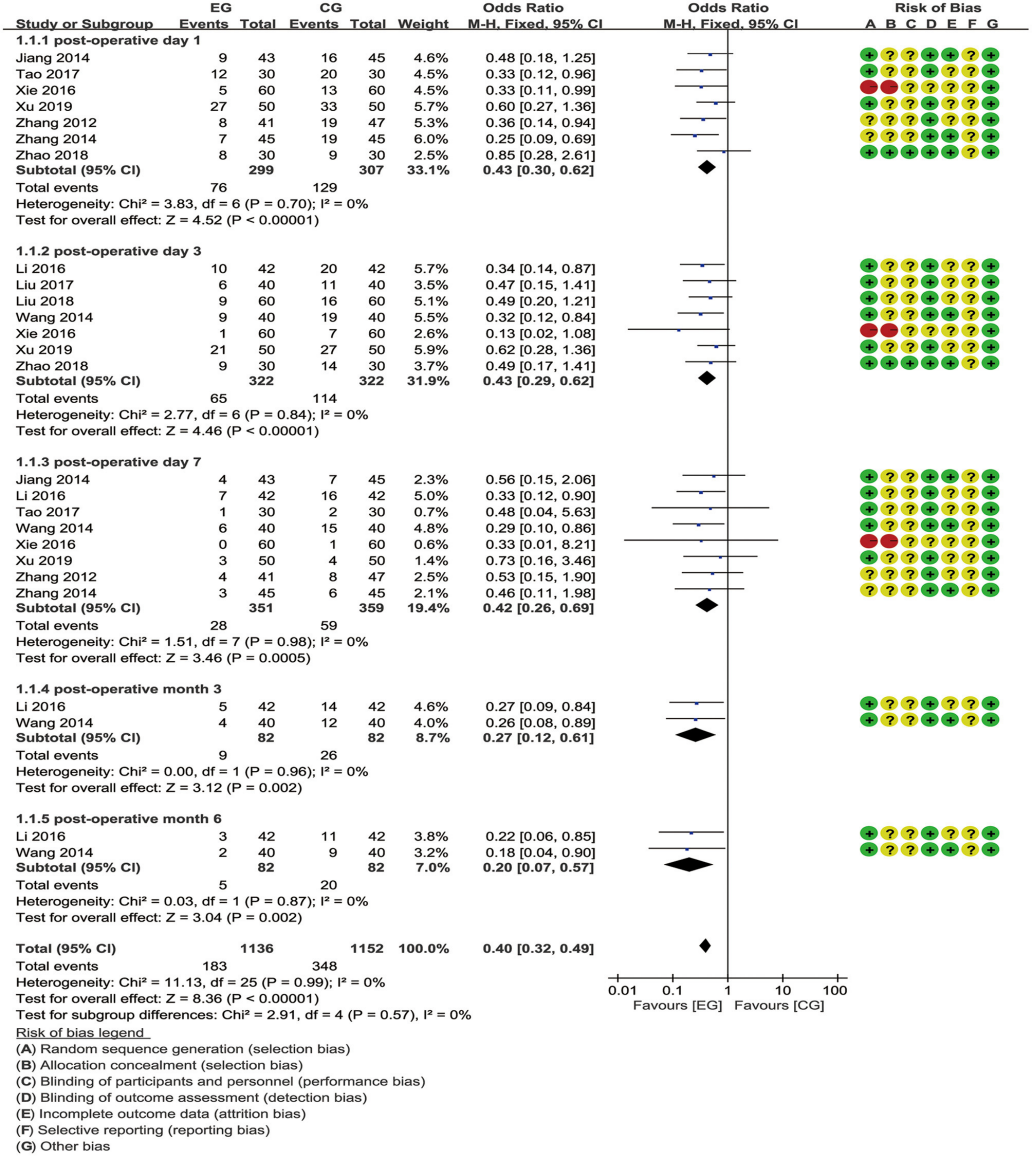
Meta-analysis and forest plot and for the incidence of postoperative cognitive dysfunction (POCD) at different periods.

The results from meta-analysis and the evidence quality of the effect of EA on POCD are given in Table 2. All the 11 included studies (17–27) reported the primary outcome. An overall meta-analysis showed moderate-certainty reduction in the incidence of POCD for EA pretreatment [OR = 0.40 (95% CI: 0.32, 0.49), *p* < 0.00001, *I*^2^ = 0%]. A further subgroup analysis was conducted to estimate the effects of EA on the incidence of POCD at 1, 3, 7 days, and 3 and 6 months after operation. For short-term outcomes, EA pretreatment significantly decreased the incidence of POCD at postoperative day 1 [OR = 0.43 (95% CI: 0.30, 0.62), *p* < 0.00001, *I*^2^ = 0%], 3 [OR = 0.43 (95% CI: 0.39, 0.62), *p* < 0.00001, *I*^2^ = 0%], and 7 [OR = 0.42 (95% CI: 0.26, 0.69), *p* = 0.0005, *I*^2^ = 0%], with no heterogeneity and a fixed-effects model. In the medium and long term, EA compared to no treatment had an obvious effect on the reduction in incidence of POCD at postoperative month 3 [OR = 0.27 (95% CI: 0.12, 0.61), *p* = 0.002, *I*^2^ = 0%] and 6 [OR = 0.20 (95% CI: 0.07, 0.57), *p* = 0.002, *I*^2^ = 0%], with no heterogeneity and a fixed-effects model (Figure 3). Most importantly, subgroup analysis by the EA treatment times between the EA group (EG) and the control group (CG) showed that the incidence of POCD was significantly lower in the EG than that in the CG, whether it is EA treatment once intraoperative [OR = 0.38 (95% CI: 0.29, 0.50), *p* < 0.00001, *I*^2^ = 0%] or multiple EA treatment in perioperative period [OR = 0.41 (95% CI: 0.29, 0.60), *p* < 0.00001, *I*^2^ = 0%] (Supplementary Figure 2). Furthermore, anesthesia method, as an important risk factor for POCD, was considered for subgroup analysis, and the result indicated that the EG presented a significant reduction in the incidence of POCD both general anesthesia [OR = 0.46 (95% CI: 0.36, 0.60), *p* < 0.00001, *I*^2^ = 0%] and spinal-epidural anesthesia [OR = 0.29 (95% CI: 0.19, 0.43), *p* < 0.00001, *I*^2^ = 0%], compared to the CG (Supplementary Figure 3).

**Table 2:** Main findings of the meta-analysis of electroacupuncture for the prevention of POCD after hip and knee arthroplasty.

**Variable**	**No. of studies**	**No. of participants**	**Effect estimate (95% CI)**	***I*** **^2^ Heteogeneity, %**	**GRADE**
**Incidence of POCD**					Low
1 d	7	606	0.43 (0.30–0.62)	0	
3 d	7	644	0.43 (0.29–0.62)	0	
7 d	8	710	0.42 (0.26–0.69)	0	
3 m	2	164	0.27 (0.12–0.61)	0	
6 m	2	164	0.20 (0.07–0.57)	0	
Treatment = 1	6	1,410	0.38 (0.29–0.50)	0	Low
Treatment >1	5	1,096	0.41 (0.29–0.60)	0	
General anesthesia	9	1,850	0.46 (0.36–0.60)	0	Low
Spinal-epidural anesthesia	2	656	0.29 (0.19–0.50)	0	
**MMSE scores**					Moderate
1 d	2	160	1.77 (−1.13 to 4.67)	94	
3 d	4	360	2.21 (1.19–3.23)	84	
**NSE**					Very Low
0 h	2	116	−0.35 (−0.69 to −0.02)	0	
24 h	2	116	−2.20 (−2.69 to −1.70)	0	
48 h	2	116	−1.23 (−1.68 to −0.77)	37	
**S-100β**					Very low
0 h	4	261	−0.08 (−0.18 to 0.02)	79	
24 h	5	321	−0.05 (−0.11 to 0.01)	59	
48 h	2	116	−0.15 (−0.36 to 0.07)	68	
72 h	2	140	−0.06 (−0.17 to 0.06)	64	
**IL-1β**					Very low
0 h	3	290	−1.72 (−3.28 to −0.16)	53	
24 h	4	350	−16.43 (−28.43 to −4.43)	99	
72 h	2	140	−31.99 (−46.04 to −17.94)	97	
**TNF-α**					Very low
0 h	3	290	−2.35 (−4.88 to 0.18)	81	
24 h	4	350	−23.47 (−38.82 to −8.12)	99	
72 h	2	140	−31.55 (−40.54 to −22.57)	95	

*GRADE, Grades of Recommendation, Assessment, Development, and Evaluation; POCD, postoperative cognitive dysfunction*.

#### Mini-Mental State Examination Scores

**Figure 4:**
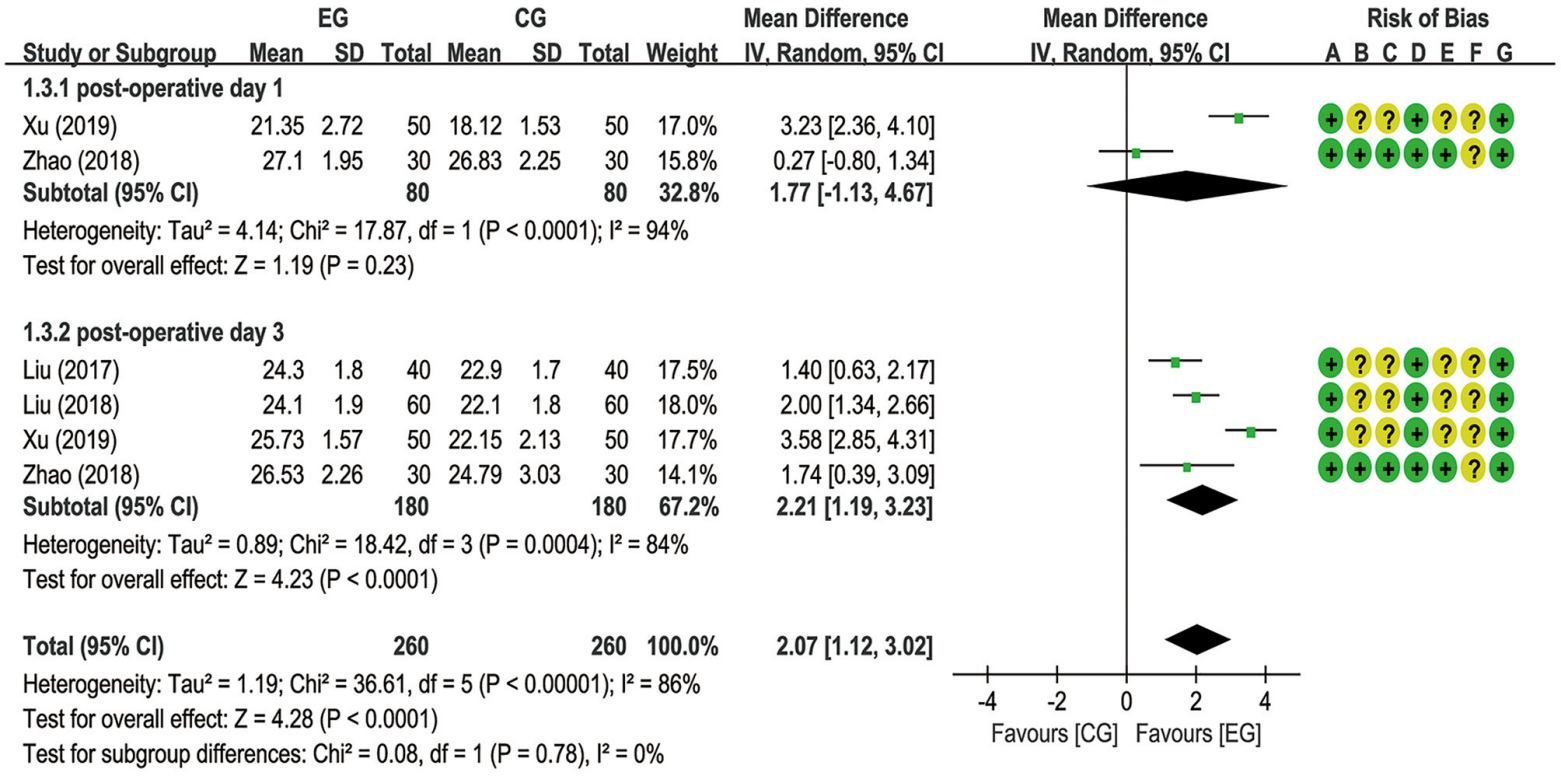
Meta-analysis and forest plot and for the Mini-Mental State Examination (MMSE) scores at different periods.

The MMSE score is one of the most common measurements for assessing cognitive function. A meta-analysis pooled from two trials (17, 18) involving 160 patients showed that there was no statistically significant difference in the improvement of the MMSE scores between the EG and the CG on the first postoperative day [MD = 1.77 (95% CI: −1.13, 4.67), *p* = 0.23, *I*^2^ = 94%], with high heterogeneity and a random-effects model. By contrast, a meta-analysis of 4 studies (17–20) revealed that, when compared to the CG, EA significantly improved the MMSE scores on the third postoperative day [MD = 2.21 (95% CI: 1.19, 3.23), *p* < 0.0001, *I*^2^ = 84%], with substantial heterogeneity and a random-effects model (Figure 4).

#### Relevant Serum Indexes Including NSE, S100β, IL-1β, and TNF-α

**Figure 5:**
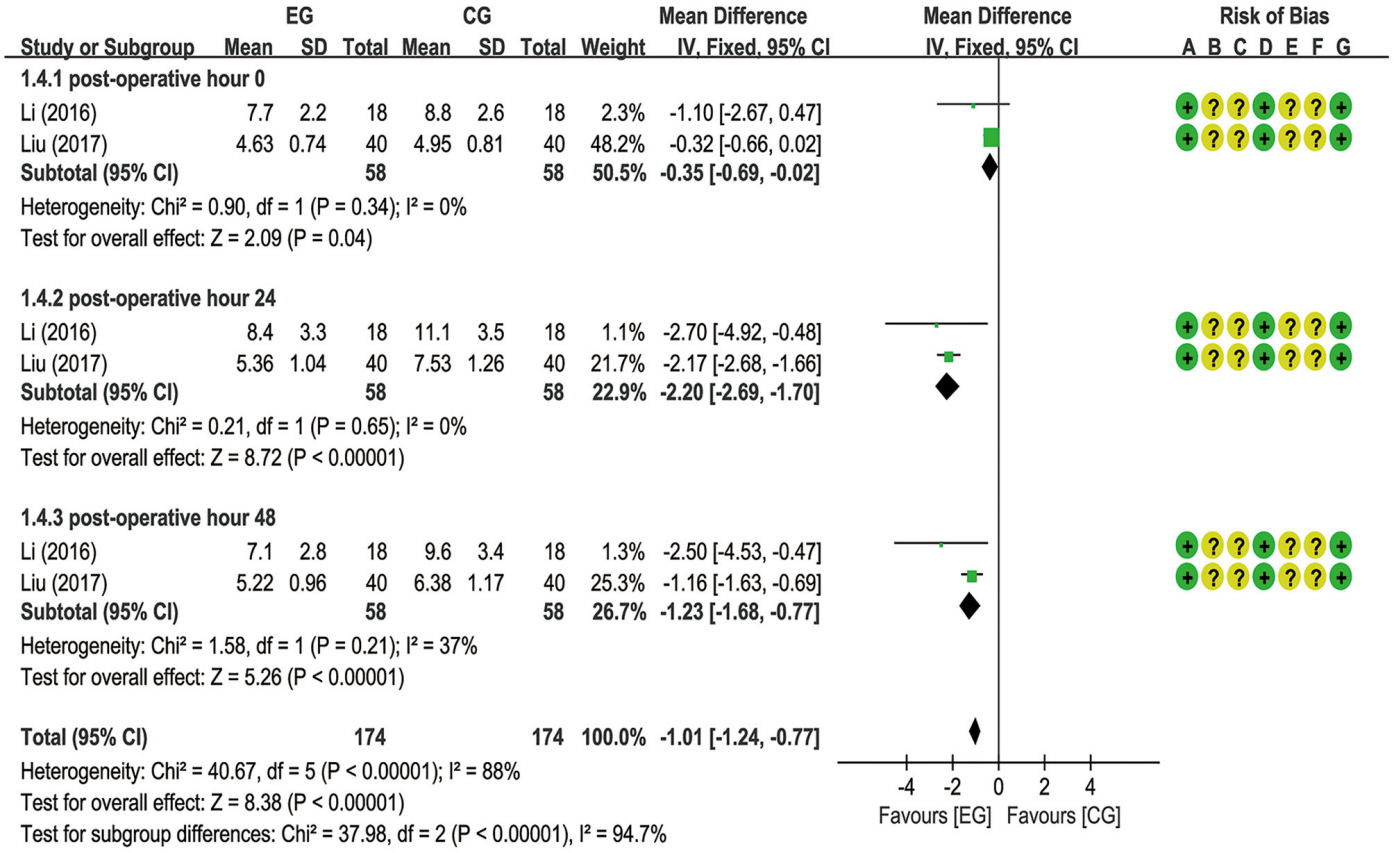
Meta-analysis and forest plot and for neuron-specific enolase (NSE) at different periods.

**Figure 6:**
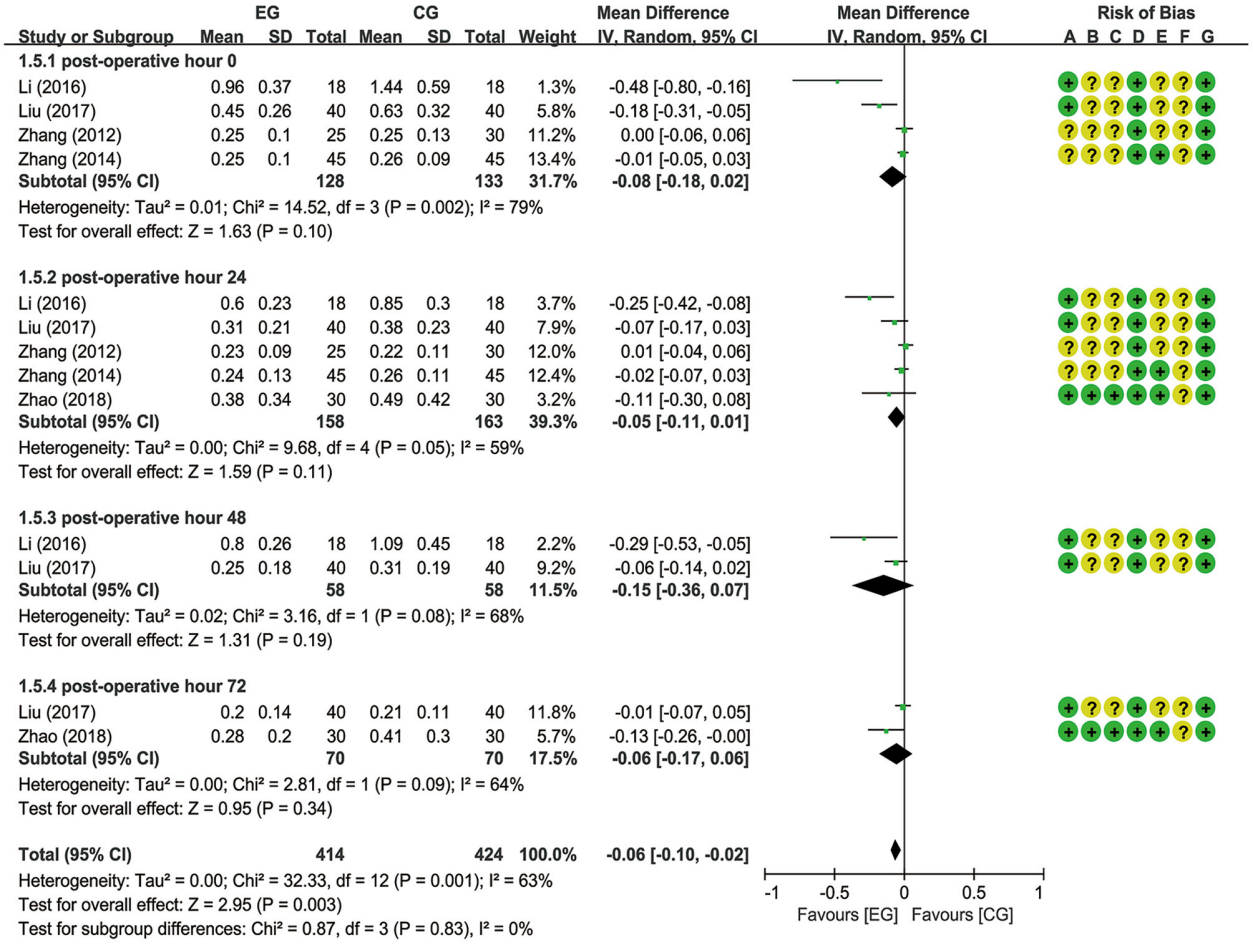
Meta-analysis and forest plot and for S100β at different periods.

**Figure 7:**
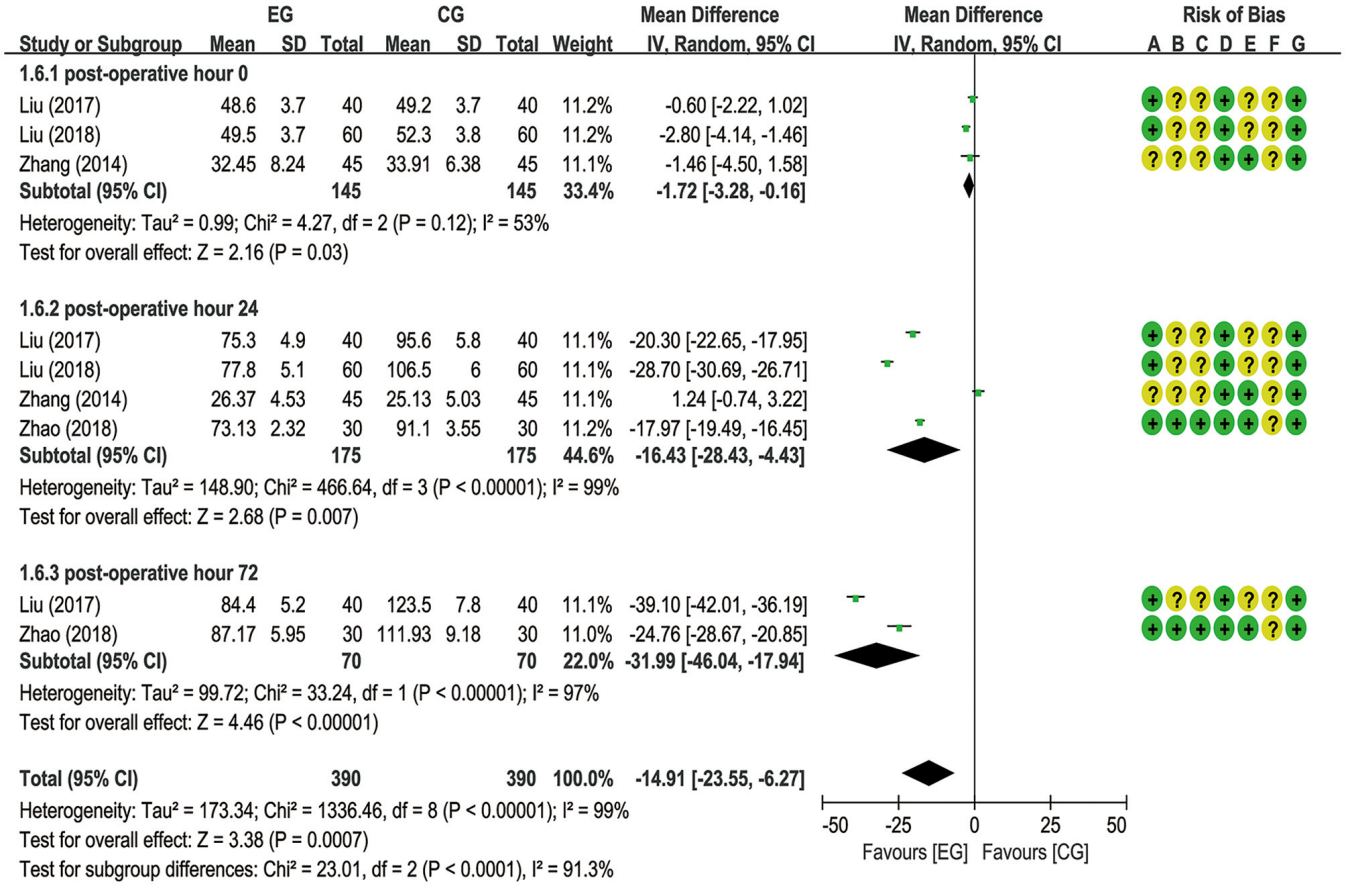
Meta-analysis and forest plot and for interleukin-1β (IL-1β) at different periods.

**Figure 8:**
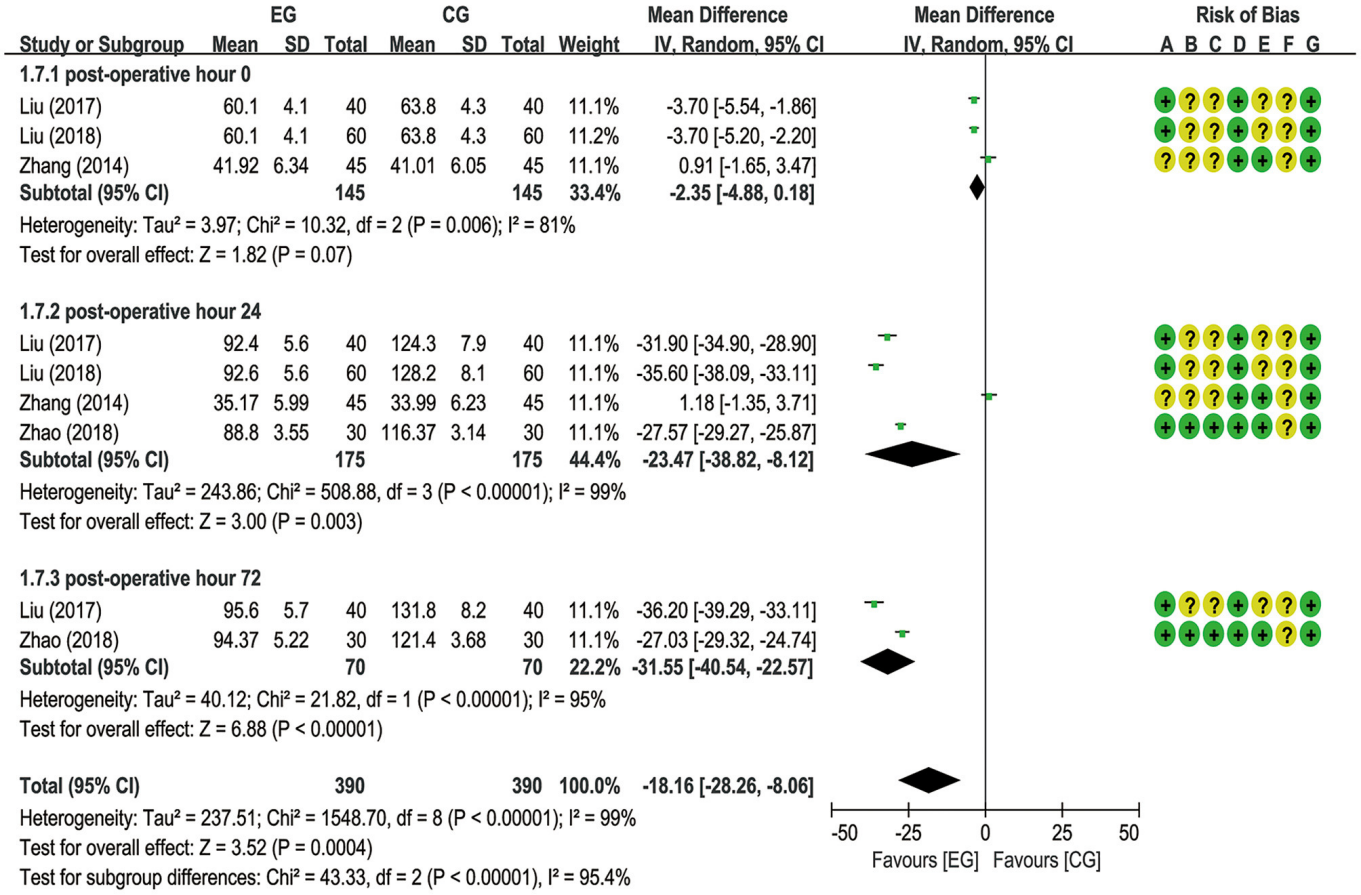
Meta-analysis and forest plot and for tumor necrosis factor-α (TNF-α) at different periods.

A total of six trials (18–20, 22, 26, 27) reported EA preconditioning for effects of serum indexes including NSE, S100β, IL-1β, and TNF-α. From a fixed-effects model, the pooled data from two trials (20, 22) suggested that EA significantly decreased the NSE expression level at postoperative hours 0 [MD = −0.35 (95% CI: −0.69 to −0.02), *p* = 0.04, *I*^2^ = 0%], 24 [MD = −2.20 (95% CI: −2.69 to −1.70), *p* < 0.00001, *I*^2^ = 0%], and 48 [MD = −1.23 (95% CI: −1.68 to −0.77), *p* < 0.00001, *I*^2^ = 37%], with low heterogeneity (Figure 5). A meta-analysis for S100β failed to demonstrate significant difference between the EG and the CG at postoperative hours 0 [MD = −0.08 (95% CI: −0.18 to 0.02), *p* = 0.10, *I*^2^ = 79%], 24 [MD = −0.05 (95% CI: −0.11 to 0.01), *p* = 0.11, *I*^2^ = 59%], 48 [MD = −0.15 (95% CI: −0.36 to 0.07), *p* = 0.19, *I*^2^ = 68%], and 72 [MD = −0.06 (95% CI: −0.17 to 0.06), *p* = 0.34, *I*^2^ = 64%] and the number of articles included in the subgroup analysis was 4 (20–27), 5 (18–27), 2 (20–22), and 2 (18–20), respectively (Figure 6). With respect to IL-1β, a meta-analysis that included 3 (19–26), 4 (18–26), and 2 studies (18–20), respectively, revealed lower IL-1β levels in the EG compared to the CG at postoperative hours 0 [MD = −1.72 (95% CI: −3.28 to −0.16), *p* = 0.03, *I*^2^ = 53%], 24 [MD = −16.43 (95% CI: −28.43 to −4.43), *p* = 0.007, *I*^2^ = 99%], and 72 [MD = −31.99 (95% CI: −46.04 to −17.94), *p* < 0.00001, *I*^2^ = 97%], with substantial heterogeneity and a random-effects model (Figure 7). With respect to TNF-α, no significant differences have been observed between the EG and the CG at postoperative hour 0 [MD = −2.35 (95% CI: −4.88 to 0.18), *p* = 0.07, *I*^2^ = 81%]. It is with a remarkable reduction in the EG in contrast to the CG at postoperative hours 24 [MD = −23.47 (95% CI: −38.82 to −8.12), *p* = 0.003, *I*^2^ = 99%] and 48 [MD = −31.55 (95% CI: −40.54 to −22.57), *p* < 0.00001, *I*^2^ = 95%], with substantial heterogeneity and a random-effects model (Figure 8).

### Sensitivity Analysis

Although subgroup analyses were done based on different observation times, the heterogeneity of some outcomes remained high (*I*^2^ = 86%, 99 and 99% for the MMSE scores, IL-1β, and TNF-α, respectively). Therefore, a method of one study excluded at a time was used to detect the source of heterogeneity and to assess whether the results could have been influenced. The results showed that there was no significant influence on the pooled MD value and the overall heterogeneities (Supplementary Table 1).

### Publication Bias

No evidence for significant publication bias was detected by Begg's test and Egger's test among the included studies (Supplementary Table 2).

### Evidence Quality Assessment

The quality of the evidence was assessed by the Grading of Recommendations, Assessment, Development, and Evaluation (GRADE) system. The summary of findings indicated that there was low certainty in the incidence of POCD, moderate certainty in the MMSE scores, and very low certainty in NSE, S100β, IL-1β, and TNF-α levels (Supplementary Figure 4).

### Adverse Events

A total of four studies (18, 22, 24, 27) paid attention to adverse events. Three of four studies (18, 22, 24) merely stated that no serious adverse events were observed in their trial. One study (27) reported in detail that the number of patients with postoperative nausea and vomiting and pulmonary infection in the EG was 5 and 4, respectively and in the CG, the number of patients with postoperative nausea and vomiting, and pulmonary infection were 6 and 5, respectively.

## Discussion

In recent years, a higher incidence of POCD has been found in elderly patients with total joint replacement. Until now, available means to prevent and cure POCD are still lacking. Some studies have demonstrated that EA is beneficial to restore the homeostatic balance of nerve function and it has been suggested to prevent POCD. In this study, we investigated the effect of EA against POCD in an elderly patient undergoing total joint replacement. We included eleven RCTs with a total of 949 patients and found that EA therapy was effective in protecting cognitive function during total joint replacement surgery in the aged patient. First, EA pretreatment significantly reduced the incidence of POCD at different observation times after surgery compared to sham EA and no treatment, and the consistent results were found in the subgroup analysis of EA treatment times and anesthesia method. Second, EA therapy was superior to no treatment in improving the MMSE scores on the third postoperative day, but not on the first postoperative day. Finally, EA decreased the levels of NSE and IL-1β at an early stage after the operation. But, no substantial effects were observed for S100β. In terms of TNF-α, EA did not show any significant improvement at postoperative hour 0, while the results reversed at postoperative hours 24 and 48. In addition, EA did not increase or decrease adverse events.

Postoperative cognitive dysfunction is associated with a variety of risk factors such as surgery-, anesthesia-, and patient-related factors. Among these, the effect of the operation type in POCD remains arguable. Previous studies showed that a higher incidence of POCD in patients undergoing cardiac surgery than in patients with non-cardiac surgery (28). Recently, Evered et al. reported that the incidence of POCD in elderly patients at 3 months was not statistically significant between total hip joint replacement and coronary artery bypass graft surgery (29). It is reported that patients over the age of 65 years who underwent non-cardiac surgery had a 26% prevalence of POCD within a few weeks, which decreased to 10% in 3 months postoperatively (30). Of the 11 trials included, there were 7 trials about total hip replacement and 2 trials about total knee replacement and the other two trials were about total hip and knee arthroplasty. So far, no studies have demonstrated a difference in the incidence of POCD between total knee replacement and total hip replacement. Besides, the anesthesia technique is a potential risk factor for POCD. In general, regional anesthesia protects cognitive function better than general anesthesia (31). As for drug selection, Li et al. suggested that propofol sedation, compared to dexmedetomidine and midazolam sedation in elderly patients, shows a significant advantage in terms of short-term POCD incidence (32). In this study, only 2 of 11 studies were performed under spinal-epidural anesthesia and the rest were conducted under general anesthesia. Moreover, the number of trials using propofol and midazolam sedation was 10 and 9, respectively.

The effectiveness of EA for cognitive improvement has been demonstrated in various clinical and animal studies. The concrete mechanism involved in EA for POCD treatment may be related to improving cerebral blood perfusion and metabolism (33), antineuroinflammation (34, 35), antioxidant stress (3, 36), and activates sympathetic nerve fibers (37). At present, neuroinflammation plays an important role in the pathophysiology of cognitive impairment. Han et al. found that EA could enhance the expression of α7-nicotinic acetylcholine receptor (α7-nAChR) and cholinergic factors and suppress neuroinflammation in the hippocampus (38). Recently, Liu et al. made a succession of in-depth studies on the neuroanatomical basis behind EA and found that EA stimulation drives sympathetic pathways in a somatotopy- and intensity-dependent manner (39). Moreover, their findings showed that EA could activate neurons expressed a specific molecular marker, and provided a neuroanatomical basis for the selectivity and specificity of acupoints in driving specific autonomic pathways (40). EA pretreatment at “Baihui” (DU20) and “Dazhui” (DU14) may increase the learning and memory ability of POCD aged mice, which is probably related to the decrease of oxidative stress and the strengthening of hippocampal antioxidant capacity (41). In the theory of acupuncture, no excellent clinical effect can be acquired without proper acupoints selection. EA stimulation at specific acupoints could produce a therapeutic effect against POCD. Lin analyzed the application of acupoints in ancient literature and found that “Baihui” (DU20), “Shenting” (DU24), “Shendao” (DU11), “Quchi” (LI11), and “Shenmen” (HT7) were the top 5 of the acupoints application frequency in cognitive dysfunction treatment (42). For this article, “Baihui” (DU20) tied with “Shenting” (DU24) for the most selection with 5 times, which was consistent with the study above. A study reported that EA at “Baihui” (DU20) can obviously improve cognitive ability damaged by sevoflurane and its mechanism may be related to activation of Shh pathway (43). Moreover, stimulating parameters of EA could affect therapeutic effects. Among the 11 studies, there were 4 studies that selected 2–10 and 2–15 Hz, respectively, and 2 studies that used 200 Hz. It is indicated that 10 Hz EA produces longer-lasting alleviation of inflammatory pain than 100 Hz and 2–10 Hz inhibit nerve injury-caused allodynia/hyperalgesia more potently than EA at 100 Hz (44).

Some limitations in this study are supposed to list. First, we did not include studies, which reported in non-Chinese or non-English languages, and all the studies were conducted in China. Second, the overall methodological quality of the included studies was low because of incorrect randomization and inadequate blinding, which reduced the validity of the analysis results. Third, inconsistency was detected in the acupoints and the EA stimulation parameters including frequency, intensity, and duration, which may contribute to high heterogeneity and affect the results. Future studies should improve the methodology quality and be consistent in acupuncture points and stimulation parameters.

## Conclusion

In this study, the preventive effect of EA for POCD was systematically reviewed and quantified. For older patients undergoing hip and knee arthroplasty, EA pretreatment has a protective effect against POCD. EA can reduce the incidence of POCD (low evidence) and improve the MMSE scores (moderate evidence). In terms of relevant serum indexes, although EA precondition could not reduce the expression of S100β (very low evidence), it significantly decreases levels of NSE (very low evidence), IL-1β (very low evidence), and TNF-α (very low evidence). However, taking into consideration the limitation of this study, more large-scale, high-quality, and good-homogeneity RCTs are warranted.

## Data Availability Statement

The original contributions presented in the study are included in the article/Supplementary Material, further inquiries can be directed to the corresponding author/s.

## Author Contributions

LO, ZS, and ZC designed this study. LO and ZC generated the search strategy and accomplished the literature search and selection. DX and DK did the data extraction. LZ and QQ evaluated the quality assessment. TZ, YH, and WH performed data analysis and evaluated the certainty of evidence. LO drafted the first manuscript. YM revised the manuscript and supervised the study. All authors have read and approved the submitted version of the manuscript.

## Funding

This study received funding from the following projects: the Scientific Research Projects of Guizhou Traditional Chinese Medicine Bureau (No. QZYY-2019-031), the National Innovation and Entrepreneurship Training Program for College Students (201910662), and the Evaluation Project for the Key Diseases of TCM Curative Effect of The Second Affiliated Hospital of Guizhou University of Chinese Medicine (GZEYK-Y[2021]5).

## Conflict of Interest

The authors declare that the research was conducted in the absence of any commercial or financial relationships that could be construed as a potential conflict of interest.

## Publisher's Note

All claims expressed in this article are solely those of the authors and do not necessarily represent those of their affiliated organizations, or those of the publisher, the editors and the reviewers. Any product that may be evaluated in this article, or claim that may be made by its manufacturer, is not guaranteed or endorsed by the publisher.
