# Supplementary material for: Electroacupuncture for the Prevention of Postoperative Cognitive Dysfunction Among Older Adults Undergoing Hip and Knee Arthroplasty: A Systematic Review and Meta-Analysis of Randomized Controlled Trials
Source: Front Med (Lausanne). 2022 Jan 4;8:778474. doi: 10.3389/fmed.2021.778474 (PMC8764307; doi:10.3389/fmed.2021.778474)
Supplement: Supplementary file 1 [file Data_Sheet_1.docx]

Supplementary Material

# The search strategy used for PubMed:

| Query | Results |
| --- | --- |
| (((Postoperative Cognitive Dysfunctions[MeSH Terms]) OR (((((((((((((((((Postoperative Cognitive Dysfunctions)) OR (Cognitive Complication, Postoperative)) OR (Cognitive Complications, Postoperative)) OR (Complication, Postoperative Cognitive)) OR (Complications, Postoperative Cognitive)) OR (Postoperative Cognitive Complication)) OR (Postoperative Cognitive Dysfunction)) OR (Cognitive Dysfunction, Postoperative)) OR (Cognitive Dysfunctions, Postoperative)) OR (Dysfunction, Postoperative Cognitive)) OR (Dysfunctions, Postoperative Cognitive)) OR (Postoperative Cognitive Dysfunctions)) OR (Postoperative Decline)) OR (Decline, Postoperative)) OR (Declines, Postoperative)) OR (Postoperative Declines))) AND (randomized controlled trial[Publication Type] OR randomized[Title/Abstract] OR placebo[Title/Abstract])) AND ((Electroacupuncture[MeSH Terms]) OR (Electroacupuncture)) | **6** |

**The search strategy used for EMBASE:**

#1 'postoperative cognitive dysfunction'/exp **1285**

#2 'cognitive complication, postoperative' OR 'cognitive complications, postoperative' OR 'complication, postoperative cognitive' OR 'complications, postoperative cognitive' OR 'postoperative cognitive complication' OR 'postoperative cognitive dysfunction' OR 'cognitive dysfunction, postoperative' OR 'cognitive dysfunctions, postoperative' OR 'dysfunction, postoperative cognitive' OR 'dysfunctions, postoperative cognitive' OR 'postoperative cognitive dysfunctions' OR 'postoperative decline' OR 'decline, postoperative' OR 'declines, postoperative' OR 'postoperative declines' **2294**

#3 'electroacupuncture'/exp **6942**

#4 ' electroacupuncture'  **8144**

#5 #1 OR #2  **2294**

#6 #3 OR #4 **8144**

#7 'randomized controlled trial'/exp OR 'controlled clinical trial'/exp OR 'randomized':ti,ab OR 'placebo':ti,ab OR 'drug therapy':lnk OR 'randomly':ti,ab OR 'trial':ti,ab OR 'groups':ti,ab

**7576398**

#8 #5 AND #6 AND #7 **8**

**The search strategy used for CINAHL:**

#1 MeSH descriptor: [Postoperative Cognitive Complications] explode all trees **13**

#2 (((((((((((((((((Postoperative Cognitive Dysfunctions)) OR (Cognitive Complication, Postoperative)) OR (Cognitive Complications, Postoperative)) OR (Complication, Postoperative Cognitive)) OR (Complications, Postoperative Cognitive)) OR (Postoperative Cognitive Complication)) OR (Postoperative Cognitive Dysfunction)) OR (Cognitive Dysfunction, Postoperative)) OR (Cognitive Dysfunctions, Postoperative)) OR (Dysfunction, Postoperative Cognitive)) OR (Dysfunctions, Postoperative Cognitive)) OR (Postoperative Cognitive Dysfunctions)) OR (Postoperative Decline)) OR (Decline, Postoperative)) OR (Declines, Postoperative)) OR (Postoperative Declines)):ti,ab,kw (Word variations have been searched) **2516**

#3 MeSH descriptor: [Electroacupuncture] explode all trees **802**

#4 (Electroacupuncture):ti,ab,kw (Word variations have been searched) **2505**

#5 #1 OR #2 **2516**

#6 #3 OR #4 **2505**

#7 #5 AND #6 **15**

**The search strategy used for CNKI:**

(SU%电针 OR SU%针刺 OR SU%针) AND (SU%术后 AND SU%认知) 79

**The search strategy used for Wanfang Data:**

主题:(电针 OR 针刺) AND 主题:(术后 AND 认知) 441

**The search strategy used for VIP**

任意字段:(电针 OR 针刺) AND 主题:(术后 AND 认知) 68

**The search strategy used for SinoMed (CBM)**

"电针"[全部字段:智能] AND "认知"[全部字段:智能] 396

# Supplementary Figure 1. The search strategy in this review.


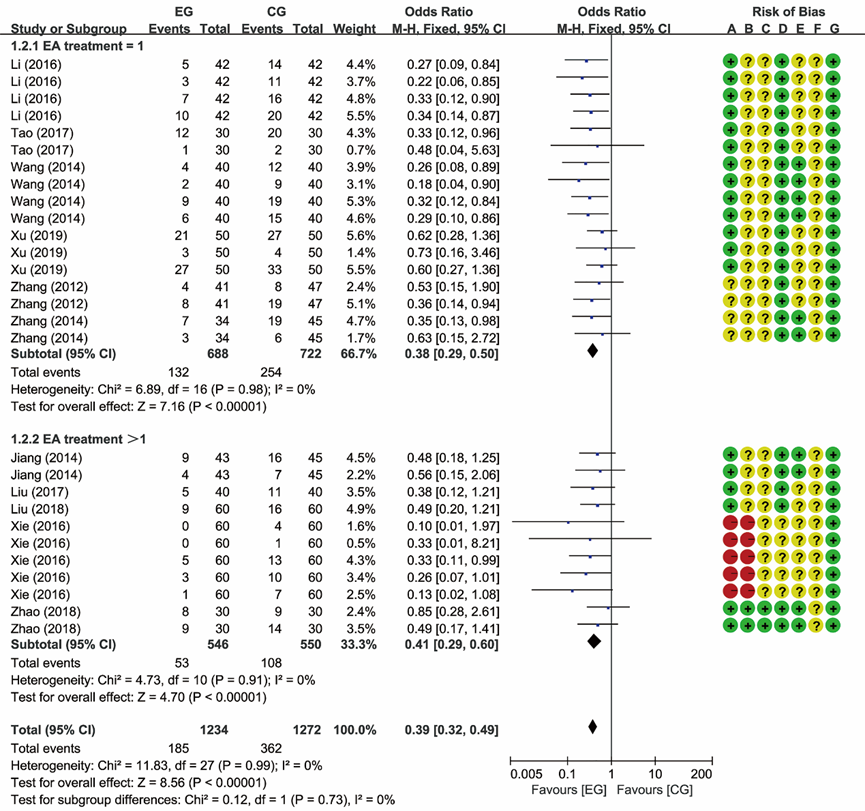


**Supplementary Figure 2.** Meta-analysis and forest plot and for the incidence of POCD at different treatment courses.


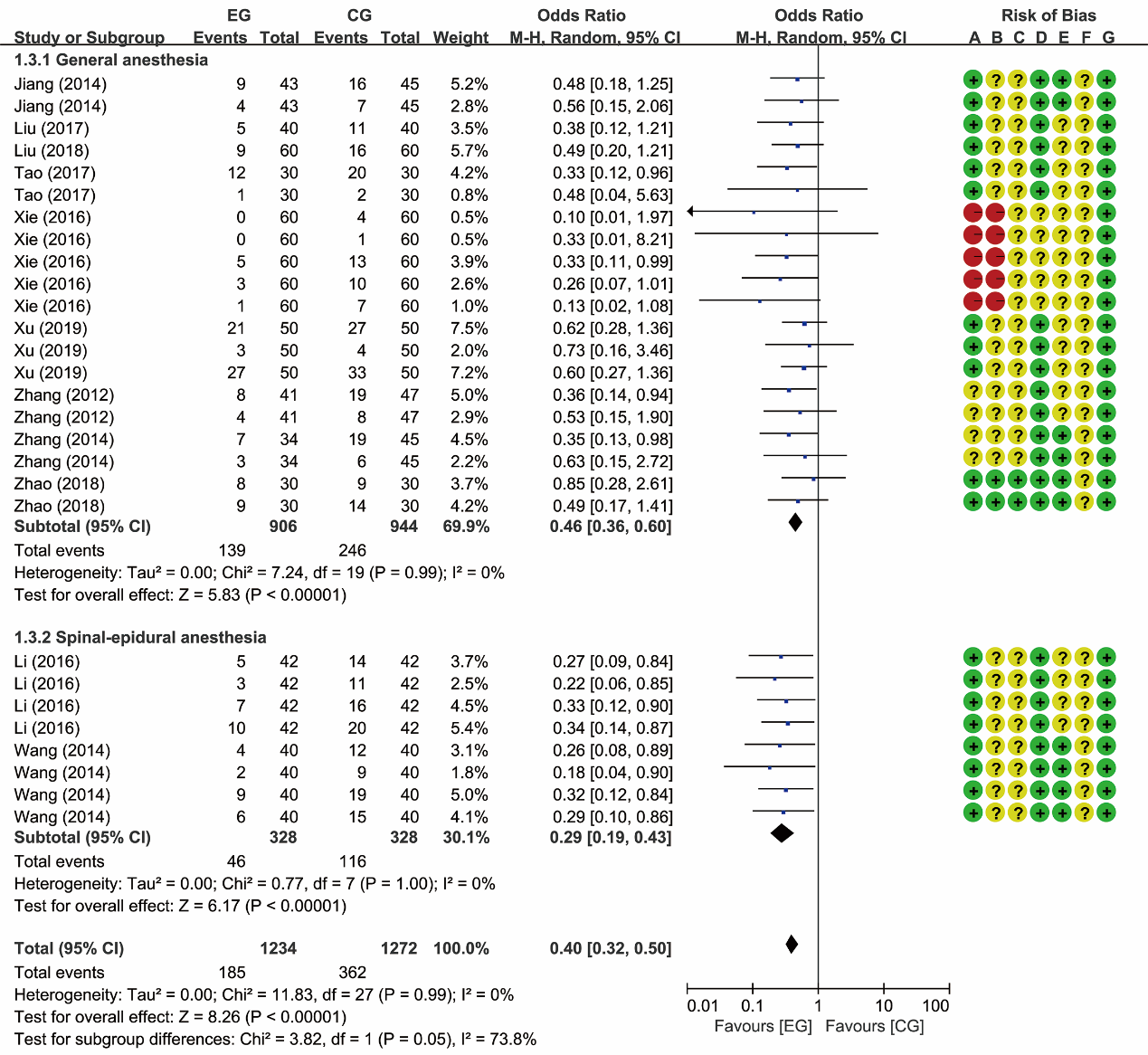


**Supplementary Figure 3.** Meta-analysis and forest plot and for the incidence of POCD at different anesthesia method.

**Supplementary Table 1.** Sensitivity analysis for MMSE scores, IL-1β, and TNF-α.

| Outcomes | References | Effect size | 95% CI | *P* | I^2^ |
| --- | --- | --- | --- | --- | --- |
| MMSE scores | Xu et al. (2019) | 2.03 | 1.66, 2.41 | < 0.00001 | 87% |
|  | Zhao et al. (2018) | 2.28 | 1.88, 2.67 | = 0.0002 | 82% |
|  | Liu et al. (2017) | 2.42 | 2.04, 2.80 | < 0.00001 | 87% |
|  | Liu et al. (2018) | 2.30 | 1.90, 2.70 | < 0.00001 | 89% |
|  | Xu et al. (2019) | 1.85 | 1.46, 2.23 | = 0.0006 | 80% |
|  | Zhao et al. (2018) | 2.25 | 1.90, 2.61 | < 0.00001 | 89% |
| IL-1β | Liu et al. (2017) | -16.71 | -26.08, -7.33 | < 0.00001 | 99% |
|  | Liu et al. (2018) | -16.43 | -26.16, -6.71 | < 0.00001 | 99% |
|  | Zhang et al. (2014) | -16.58 | -25.88, -7.28 | < 0.00001 | 99% |
|  | Liu et al. (2017) | -14.23 | -23.66, -4.81 | < 0.00001 | 99% |
|  | Liu et al. (2018) | -13.17 | -21.72, -4.62 | < 0.00001 | 99% |
|  | Zhang et al. (2014) | -16.93 | -26.12, -7.74 | < 0.00001 | 99% |
|  | Zhao et al. (2018) | -14.53 | -24.47, -4.58 | < 0.00001 | 99% |
|  | Liu et al. (2017) | -11.89 | -19.95, -3.82 | < 0.00001 | 99% |
|  | Zhao et al. (2018) | -13.69 | -22.86, -4.53 | < 0.00001 | 99% |
| TNF-α | Liu et al. (2017) | -19.97 | -31.04, -8.91 | < 0.00001 | 99% |
|  | Liu et al. (2018) | -19.98 | -30.96, -9.00 | < 0.00001 | 99% |
|  | Zhang et al. (2014) | -20.54 | -31.18, -9.90 | < 0.00001 | 99% |
|  | Liu et al. (2017) | -16.45 | -27.12, -5.78 | < 0.00001 | 100% |
|  | Liu et al. (2018) | -15.98 | -26.23, -5.73 | < 0.00001 | 99% |
|  | Zhang et al. (2014) | -20.58 | -31.19, -9.97 | < 0.00001 | 99% |
|  | Zhao et al. (2018) | -16.98 | -27.97, -6.00 | < 0.00001 | 99% |
|  | Liu et al. (2017) | -15.92 | -26.33, -5.50 | < 0.00001 | 99% |
|  | Zhao et al. (2018) | -17.05 | -28.05, -6.06 | < 0.00001 | 100% |

**Supplementary Table 2.** Assessment of publication bias.

| Outcomes | N | Begg' s test | Egger's test |
| --- | --- | --- | --- |
| Incidence of POCD (1 d) | **7** | 0.548 | 0.590 |
| Incidence of POCD (3 d) | **7** | 0.072 | 0.050 |
| Incidence of POCD (7 d) | 8 | 0.711 | 0.425 |
| Incidence of POCD (3 m) | 2 | 1 | NA |
| Incidence of POCD (6 m) | 2 | 1 | NA |
| MMSE scores (1 d) | 2 | 1 | NA |
| MMSE scores (3 d) | 4 | 1 | 0.815 |
| NSE (0 h) | 2 | 1 | NA |
| NSE (24 h) | 2 | 1 | NA |
| NSE (48 h) | 2 | 1 | NA |
| S-100β (0 h) | 4 | 0.089 | 0.065 |
| S-100β (24 h) | 5 | 0.462 | 0.057 |
| S-100β (48 h) | 2 | 1 | NA |
| S-100 (72 h) | 2 | 1 | NA |
| IL-1β（0 h） | 3 | 1 | 0.749 |
| IL-1β（24 h） | 4 | 0.734 | 0.998 |
| IL-1β（72 h） | 2 | 1 | NA |
| TNF-α（0 h） | 3 | 0.296 | 0.26 |
| TNF-α （24 h） | 4 | 1 | 0.868 |
| TNF-α （72 h） | 2 | 1 | NA |

N: number of studies; NA: not available.


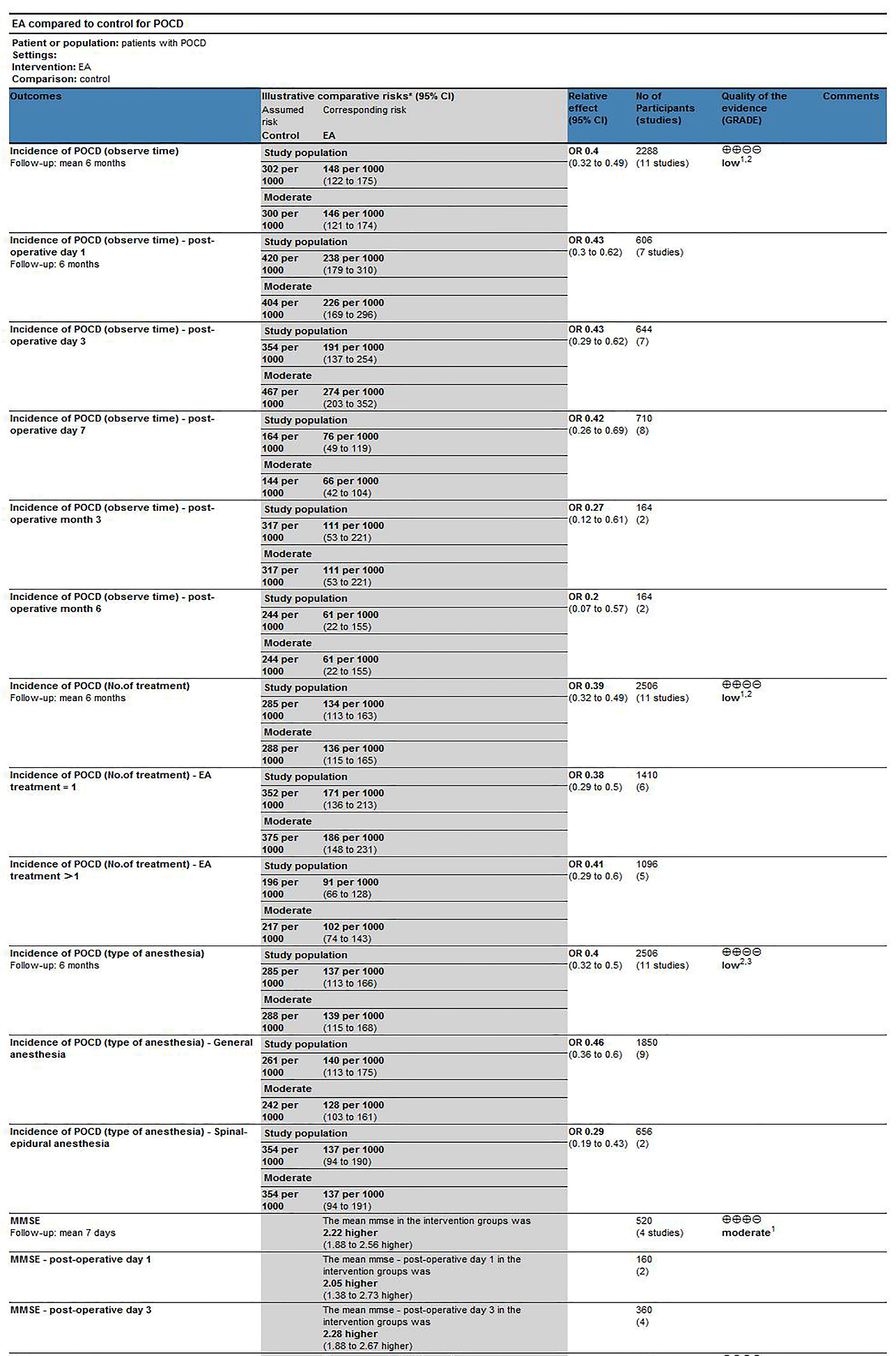


(Continued)

(Continued)


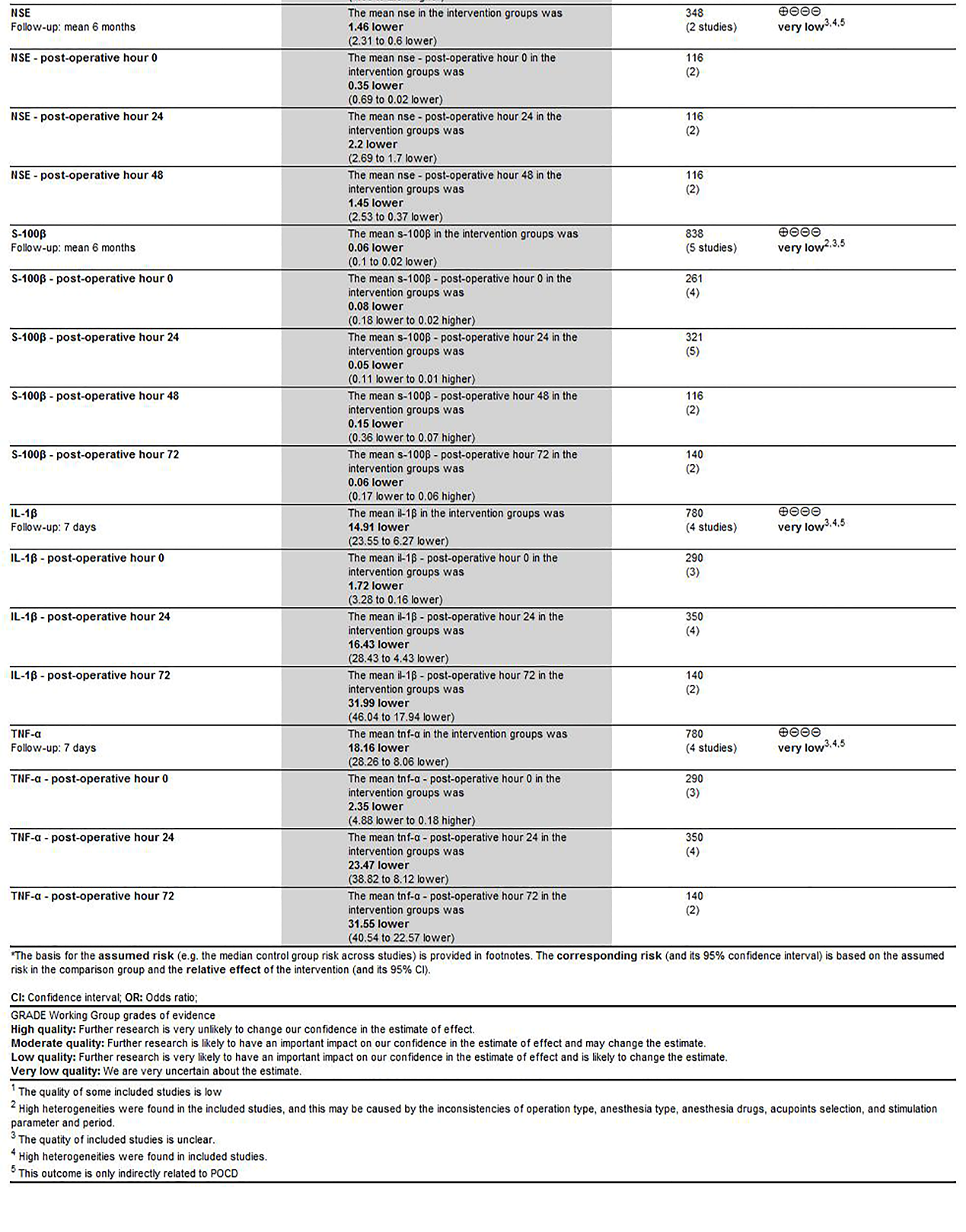


**Supplementary Figure 4.** Summary of findings table.
